# Solvent exchange in preformed photocatalyst-donor precursor complexes determines efficiency†
†Electronic supplementary information (ESI) available. See DOI: 10.1039/c7sc04533f


**DOI:** 10.1039/c7sc04533f

**Published:** 2017-12-21

**Authors:** Laura M. Kiefer, Kevin J. Kubarych

**Affiliations:** a Department of Chemistry , University of Michigan , Ann Arbor , MI 48109 , USA . Email: kubarych@umich.edu

## Abstract

In homogeneous photocatalytic reduction of CO_2_, it is widely assumed that the primary electron transfer from the sacrificial donor to the catalyst is diffusion controlled, thus little attention has been paid to optimizing this step. We present spectroscopic evidence that the precursor complex is preformed, driven by preferential solvation, and two-dimensional infrared spectroscopy reveals triethanolamine (donor)/tetrahydrofuran (solvent) exchange in the photocatalyst's solvation shell, reaching greatest magnitude at the known optimal concentration (∼20% v/v TEOA in THF) for catalytically reducing CO_2_ to CO. Transient infrared absorption shows the appearance of the singly reduced catalyst on an ultrafast (<70 ps) time scale, consistent with non-diffusion controlled electron transfer within the preformed precursor complex. Identification of preferential catalyst–cosolvent interactions suggests a revised paradigm for the primary electron transfer, while illuminating the pivotal importance of solvent exchange in determining the overall efficiency of the photocycle.

## Abstract 2

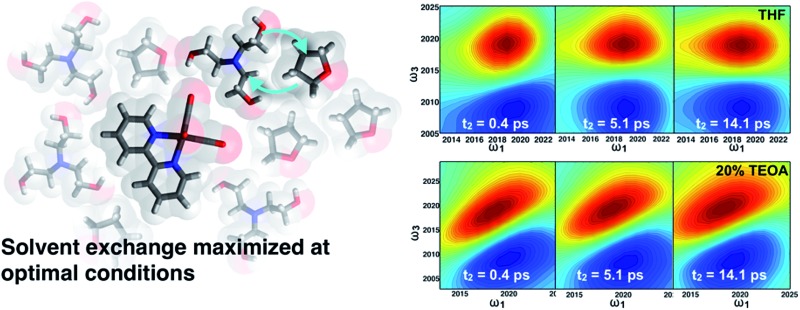
Ultrafast two-dimensional infrared spectroscopy reveals that solvent exchange reaches a maximum at ideal composition for photocatalytic reduction of CO_2_.

## Introduction

Understanding catalytic reaction mechanisms is central to optimizing selectivity and efficiency. Fundamental processes including solvation, electron transfer, and diffusion certainly occur, but their specific contributions are often obscured using common experimental techniques. Photocatalysis is further complicated by the involvement of at least one excited electronic state, but the optical excitation can synchronize events in the photocycle, simplifying elucidation of the earliest processes. Photoredox catalysis incorporates both light absorption and intermolecular charge transfer steps in addition to substrate binding and product release, so lessons learned in a given system should be generalizable.

**Fig. 1:**
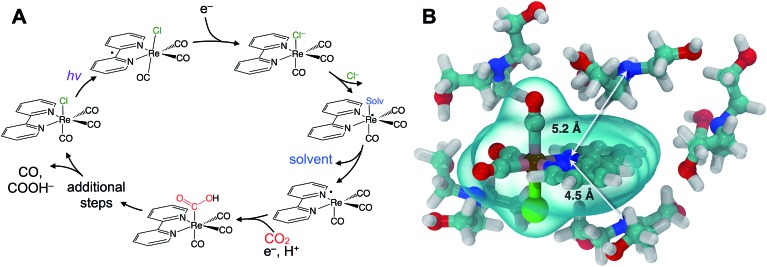
Rhenium bipyridyl photocatalysts for CO_2_ reduction to CO and COOH^–^. (A) The photocatalytic cycle starts with absorption of near-UV light, producing a metastable (∼60 ns) ^3^MLCT state that is reduced by electron transfer from an amine sacrificial donor. A solvent (or co-solvent) molecule substitutes the chloride and then dissociates leaving a radical, which subsequently binds CO_2_ as a carboxylic acid. Further downstream steps lead to final production of CO or COOH^–^. (B) Force-field optimized cluster of Re(bpy)(CO)_3_Cl with five TEOA molecules, showing the donor–acceptor distance of roughly 5 Å.

We describe the structural dynamics of a rhenium complex, Re(bpy)(CO)_3_Cl (bpy = bipyridine), an effective CO_2_ reduction photocatalyst, in solution conditions similar to those used to produce CO or COOH^–^ from CO_2_.^1^–^3^ This family of complexes, first identified by Lehn *et al.*,^1^ is generally thought to catalyze the 2-electron/2-proton reduction of CO_2_ through the sequence of steps outlined in Fig. 1A.^4^–^6^ Near-UV light (∼400 nm) excites the catalyst to a singlet charge transfer state (^1^CT) state, which relaxes through a rapid (∼0.2–1.0 ps) intersystem-crossing to a triplet metal-to-ligand charge transfer (^3^MLCT) state.^7^,^8^ Reduction by a sacrificial electron donor facilitates loss of the axial halide ligand, and the solvent or the electron donor itself coordinates to the Re center.^9^ The CO_2_ replaces the solvent (or donor) ligand, and there is some evidence that formation of a binuclear, CO_2_-bridged dimer facilitates cleavage of a C–O bond.^2^,^10^,^11^

Although this sequence of steps is supported by considerable evidence, key aspects of the molecular details are assumed without direct experimental support. In particular, the first reduction by the sacrificial donor is thought to take place following the formation of a precursor complex by diffusing through the solution. There is little reason to question this assumption, which is a basic ingredient in outer-sphere, intermolecular electron transfer.^12^,^13^ The long (∼60 ns) lifetime of the activated ^3^MLCT state permits relatively long-range diffusion before deactivation. The results we show here indicate that the precursor complex is actually preformed due to significant preferential interaction between the polar catalyst and donor amine. Preferential solvation is always a possibility in bimolecular reactions in solution, but it has not been previously described or considered for this important rhenium photocatalyst. A simple force field optimization indicates that van der Waals contact between TEOA and the rhenium complex places the donor and acceptor within ∼5 Å (Fig. 1B). We find evidence that the intrinsic ET time scale may be speed-limited by the distance dependence of electron tunneling. These findings suggest that the catalyst's efficiency could potentially be improved by increasing the yield of productive ET events.

Two-dimensional infrared (2D-IR) spectroscopy correlates excited and detected vibrational transitions, enabling decomposition of complex spectral bands into contributions from homogeneous dephasing and inhomogeneous frequency distributions arising from variations in the local solvent environment.^14^ The key observable is the time dependent loss of frequency correlation due to stochastic sampling of the frequencies within an inhomogeneously broadened band, which is known as spectral diffusion.^15^ The decay of the frequency fluctuation correlation function (FFCF, *C*(*t*) = *δω*(0)*δω*(*t*)) is the principal observable used to characterize the solvation structure and dynamics of the rhenium photocatalyst.

The FFCF offers a window into the solvation dynamics of the photocatalyst solute, revealing the time scale for solvent shell fluctuations. For a ternary mixture of the catalyst and two solvents, there is the possibility to observe the exchange of dissimilar species in the solvation shell of the vibrationally probed solute. Since solvent exchange is generally slower than the typical time scales of short range (*i.e.* librational) solvent motion, spectral diffusion can be slower in a mixture of solvents than in either solvent alone.^16^ This exchange-induced slowdown of spectral dynamics has been observed in numerous time-resolved fluorescence studies of solvent mixtures, and we have identified the dynamical signature in 2D-IR spectroscopy.^17^,^18^

## Results and discussion

**Fig. 2:**
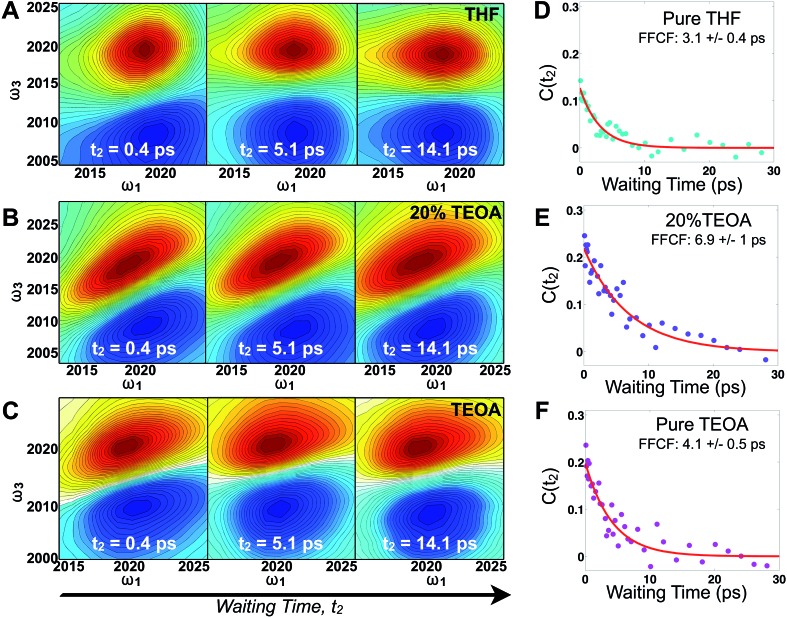
2D-IR spectra of Re(bpy)(CO)_3_Cl in THF/TEOA solutions. Absorptive 2D-IR spectra of the A′(1) CO stretching band in (A) THF, (B) 20% (v/v) TEOA in THF, (C) TEOA at three waiting times (0.4, 5.1 and 14.1 ps) illustrating the changes due to solvation dynamics. The *x*-axis, *ω*_1_, represents the infrared excitation (pump) frequencies in cm^–1^, and the *y*-axis, *ω*_3_, represents the infrared detection (probe) frequencies in cm^–1^. The vibrational transition *v* = 0 → 1 is shown in red and *v* = 1 → 2 is in blue. The inhomogeneous width is maximal in mixed solvent, and full decays of the FFCF (D–F) indicate that spectral fluctuations are slowest in the mixed solvent due to dissimilar solvent exchange.

Fig. 2 displays absorptive 2D-IR spectra of the totally symmetric A′(1) carbonyl vibrational stretching mode (2019 cm^–1^) of Re(bpy)(CO)_3_Cl at distinct waiting times (*t*_2_ = 0.4, 5.1, and 14.1 ps) of the Re complex in pure tetrahydrofuran (THF), 20% triethanolamine (TEOA) in THF, and pure TEOA, highlighting the differences between the environments. The absorptive spectra depict both the *v* = 0 → *v* = 1 (red, top) as well as the *v* = 1 → *v* = 2 (blue, bottom) transitions. All analyses are performed on the fundamental *v* = 0 → *v* = 1 transition. At early waiting times, it is clear that the Re complex in pure THF has very little inhomogeneous broadening based on the relatively narrow diagonal width. The solvent mixture induces a noticeable increase in inhomogeneous broadening, reflecting the increased diversity of solvent environments. The FFCF indicates that in the 20%/80% TEOA/THF solution, frequency correlation persists even at longer waiting times compared with either of the two pure solvent cases.

**Fig. 3:**
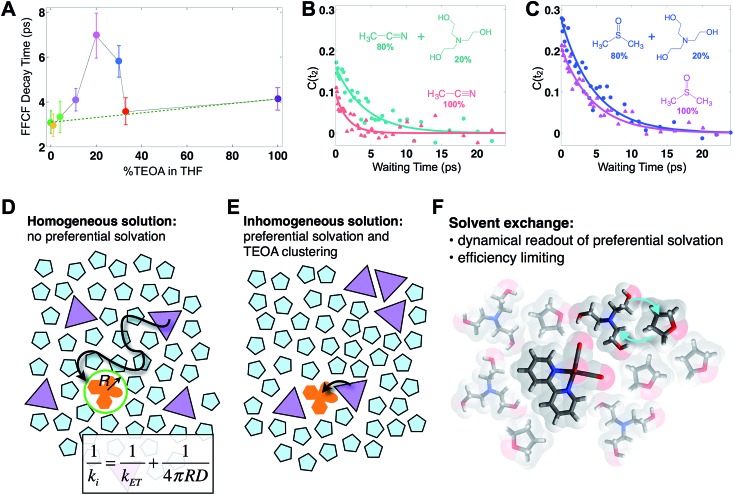
Solvent composition dependence. (A) Exponential time constants for spectral diffusion of the Re(bpy)(CO)_3_Cl symmetric CO stretch in various compositions of TEOA in THF, ranging from 0 to 100% (v/v). Spectral diffusion is slowest at 20%, which corresponds to the maximal degree of solvent exchange. 20% composition yields the highest efficiency of CO production in active photocatalytic reactors.^38^,^39^ (B) 20% TEOA in acetonitrile also shows a pronounced slowdown, whereas (C) in DMSO, there is no significant composition dependence. In DMSO, Re(bpy)(CO)_3_Cl does not display evidence for preferential solvation by TEOA. Cartoons depicting (D) a homogeneous solution where the primary charge transfer would be expected to be diffusion controlled; (E) an inhomogeneous solution where preferential solvation and co-solvent clustering alter the local concentration; (F) dissimilar co-solvent exchange in the first solvation shell of the catalyst.

In this series of experiments, we observe the A′(1) mode's spectral dynamics as a function of fraction of TEOA in THF (Fig. 3A). At low TEOA concentrations, there are gradual increases in the FFCF decay time relative to neat THF (3.1 ± 0.5 ps). As the composition nears 20% TEOA (v/v), the correlation decay time increases to a maximum of 6.9 ± 1 ps. At slightly higher TEOA concentrations, spectral diffusion becomes faster, ultimately reaching 4.1 ± 0.5 ps in pure TEOA.

The spectral diffusion time constants of the mixtures depend on the relative contributions of fast libration-like solvent dynamics and the slower solvent exchange (Fig. 3F). Since we fit our FFCF decays to single exponentials with constant offsets, we measure a time scale that effectively mixes the two dynamical contributions. We have previously shown in water/dimethylformamide solutions that the solvent exchange time scale is predictable from mutual diffusion of the two solvents.^16^ The time scales reported here are consistent with the solvent exchange mechanism (discussion in ESI†). The quantitative time scales are not intrinsically important, rather we view the introduction of a slow component as a signature of solvent exchange.^17^,^18^ Absent preferential interactions, exchange of dissimilar solvent species should be maximal at 50% mole fraction in analogy to the maximal entropy of mixing at equal concentrations. Preferential solvation, on the other hand, shifts the maximum towards lower concentration of the preferred species. We expect TEOA to preferentially solvate the highly polar (14.1 Debye)^19^,^20^ Re catalyst because TEOA is more polar than THF. We note that the spectral diffusion time scales change with increased concentration above 20% TEOA, and are anti-correlated with the solution's viscosity. Indeed, the viscosity of TEOA is two orders of magnitude higher than THF, yet we find very similar dynamical time scales in the two neat solvents. Decoupling from viscosity is not unusual, however, and has been seen in several contexts such as simple liquids, glass forming liquids, and liquid crystals.^19^,^21^–^24^

To determine whether the composition dependent line shapes and dynamical slowdown are unique to THF, we performed 2D-IR experiments of Re(bpy)(CO)_3_Cl in 20%/80% TEOA/DMSO and in 20%/80% TEOA/CH_3_CN and compared these results to those in the respective pure solvents (Fig. 3B and C). The correlation decay times of the rhenium complex in the solvent mixtures exhibit a slowdown in CH_3_CN (pure: 1.7 ± 0.3 ps; mixture: 4.2 ± 0.7 ps), but not in DMSO (pure: 4.5 ± 0.4 ps; mixture: 4.7 ± 0.5 ps). These results are consistent with the picture that emerges from the TEOA/THF data: we expect inhomogeneous (Fig. 3E), preferential solvation by TEOA in CH_3_CN, but not in DMSO, where we anticipate a largely homogeneous solution (Fig. 3D). This expectation is based on spectroscopic studies using a solvation probe dye (betaine-30), as discussed in the ESI.† Similar to the comparison of the diagonal line widths of the absorptive 2D spectra, the initial value of the FFCF, *C*(*t*_2_ = 0), is related to the inhomogeneity of the band.^25^ We find that in all cases the solvent mixtures are more inhomogeneously broadened than the pure solvents reflected by the larger initial FFCF values.

**Fig. 4:**
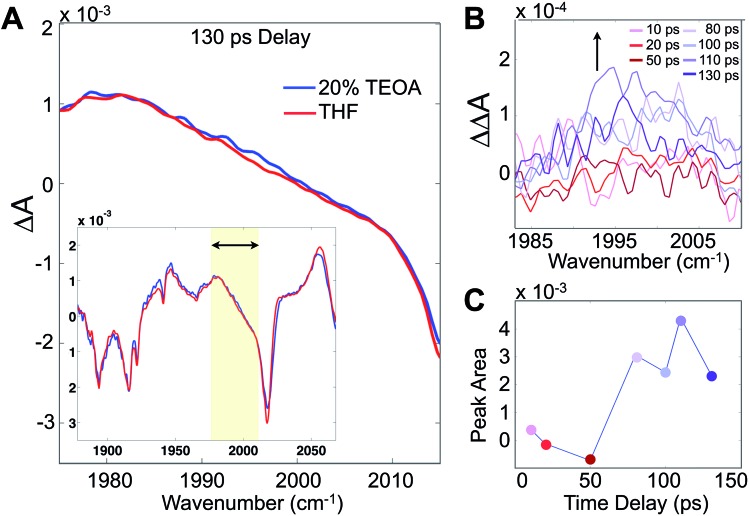
Transient IR absorption tracks the appearance of the singly reduced species. (A) Differential absorption (pump on – pump off) of Re(bpy)(CO)_3_Cl in 20% TEOA/THF solution in the carbonyl stretching band (inset), and zoomed to the region around 1996 cm^–1^, corresponding to the singly reduced species. (B) Double-difference spectra at various time delays between the 400 nm pump and mid-IR probe showing the growth of the band. (C) Integrated singly-reduced band indicates a growth on a ∼70 ps time scale, which is significantly faster than expected by diffusion. The standard deviations of the double difference signals in (B) are in the range of 1.5–3.6 × 10^–5^ OD.

Although these 2D-IR results and the remarkably clear slowdown of spectral diffusion indicate that the TEOA sacrificial donor preferentially solvates the Re photocatalyst, definitive mechanistic insights must link the solvent structure to the catalytically essential primary electron transfer event. There have been many studies of Re photophysics using time-resolved IR spectroscopy as well as extensive spectroelectrochemical investigations,^26^,^27^ but to-date there have been no ultrafast (*i.e.* sub-ns) transient IR absorption measurements of the photoinduced reduction by a sacrificial donor. The photophysics in the absence of the donor yields a substantial background, but following the initial 10–20 ps attributed to solvation and vibrational cooling of the ^3^MLCT state, there is no significant dynamical evolution of the transient spectra. To isolate the reduced species, we employ a careful double-difference method, where we measure transient IR absorption spectra in the presence and absence of the TEOA donor using a flowing cell, leaving the beam alignment completely unchanged for the two samples. This approach enables us to measure the very small, ∼50 μOD, differences attributable to the weakly absorbing singly reduced species (Fig. 4A). The IR transitions of the reduced photocatalyst have been identified with spectroelectrochemistry, though with no information about the time dependence of its formation.^28^,^29^

Integrating the double-difference spectra (*i.e.* ΔΔ*A*) in the vicinity of 1996 cm^–1^, characteristic of the singly reduced species (Fig. 4B) [Re(bpy)(CO)_3_Cl]˙^–^,^28^ gives a signal that first appears between 50 and 80 ps following the 400 nm excitation. Taking the midpoint 65 ps to be a measure of the ET time scale (Fig. 4C), we can compare with a diffusion controlled prediction. According to the Collins–Kimball treatment of diffusion controlled electron transfer (see full discussion in the ESI†),^30^ given our sample conditions and our experimentally measured diffusion constant of TEOA in THF solution, the fastest expected time scale for the ET reaction is ∼350 ps. Including a finite time scale intrinsic ET transfer rate, as well as steric and orientational contributions, the true diffusion controlled time scale must be considerably slower than this limiting value. Hence, our measured ET time scale is at least an order of magnitude faster than would be anticipated based on a diffusion controlled process, indicating that the TEOA must be in close proximity to the rhenium complex. Our result is generally consistent with a similar transient IR absorption study of Re(bpy)(CO)_3_Br acting as a photosensitizer for H_2_ production found prompt reduction of the Re complex, followed by slow, diffusion controlled electron transfer to the cobalt catalyst.^31^

It is well established that quenching of excited states by electron transfer can occur on a range of time scales due to at least two distinct pathways.^12^,^32^ Any ternary system of donor, acceptor and solvent will have an instantaneous spatial distribution of species giving rise to a distribution of microscopic quenching events. An ensemble measurement senses the overall kinetics, though in some instances it is possible to spectroscopically tag subsets of the ensemble by tuning the optical excitation.^33^ A second phase of kinetics arises when donors and acceptors diffusively encounter each other. When there is a significant difference in the “intrinsic” ET time scale (initial static distribution) and the generally much slower diffusion limited reaction, expressions such as those derived by Collins and Kimball (Fig. 3D) can describe the overall observed reaction rates.^30^

The transient IR difference spectra provide support for an ultrafast phase of electron transfer that occurs between the preferentially solvated TEOA electron donors and the Re photocatalyst. Using the tunneling picture of electron transfer of Gray *et al.*, our 50–80 ps ET time scale would put the TEOA within 7–9 Å of the rhenium complex.^34^ Simple force field optimized geometries are consistent with this estimate, supporting the picture of the TEOA and the Re catalyst in van der Waals contact (Fig. 1B). There is evidence for preferential interaction between the rhenium bipyridyl photocatalyst and TEOA in the context of a hybrid system bound to TiO_2_ nanoparticles showing fast (ps–ns) electron transfer from the TEOA donor to the TiO_2_-bound Re complex.^35^ Although our experimental observations have captured this ultrafast electron transfer event due to the preferentially solvating TEOA molecules, it is clear that not all of the ET processes occur *via* this ultrafast phase, as other measurements have shown a significant diffusive component on much slower time scales.^31^

## Conclusion

Rhenium bipyridyl complexes are currently the most effective known homogeneous photocatalysts for CO_2_ reduction.^2^,^3^,^36^ They notably combine the light absorbing photosensitizer with the catalytic center, reducing unproductive loss channels that are inevitable in multisite photosensitizer/catalyst constructs. Compared with other popular photocatalysts such as [Ru(bpy)_3_]^2+^, and [Ir(ppy)_2_(bpy)]^+^, the highly asymmetrical rhenium catalysts have large dipole moments. It is generally accepted that photocatalysts and photosensitizers must have long triplet state lifetimes so that charge transfer can occur before relaxation to the ground state. Nevertheless, Re-bpy complexes are more effective at oxidizing electron donors such as TEOA than is [Ru(bpy)_3_]^2+^ despite the Re complex's 10–100 fold shorter excited state lifetime.^37^ Though some of this oxidizing ability is due to differences in thermodynamic driving force, our results suggest that preforming the precursor complex by virtue of the preferential solvation may be a contributing factor. This alternative paradigm for photoredox catalysis provides guidance for tailoring the photocatalyst to the specific electrostatic nature of the substrates or other reagents. For the present case of photocatalytic reduction of CO_2_, our findings suggest that the reaction performance may be limited by the actual electron transfer from the TEOA donor to the Re to generate the one-electron reduced species.

Preferential solvation is an essentially structural aspect of the photocatalytic process that nevertheless has been identified using a dynamical measurement since the natural timescale for solvent exchange is too fast to be observed using, for example, NOESY NMR. What is most striking about our observations is the correlation between the picosecond time scale solvent shell dynamics and the much slower overall catalytic reaction cycle. The solution composition where we find maximum solvent exchange coincides with the optimal conditions (*i.e.* those that result in optimum quantum efficiency) for CO production.^38^,^39^ This experimental link between catalytic activity and maximal solvent exchange is consistent with the mechanistic step where the solvent or the donor coordinates to Re, but elevates the importance of the apparently rate determining dynamics of access to the catalyst. In that sense, the overall composite sequence of reduction by the donor and solvent/donor coordination is indeed diffusion controlled, but only the second process is actually diffusive. Since diffusion is essentially uncontrollable, our findings provide information necessary for catalyst optimization based on the specific sequence of molecular dynamics events, rather than the inevitably convoluted picture provided by kinetics.

## Methods

The 2DIR experiments use three mid-infrared pulses (500 nJ, ∼100 fs) to generate a third-order nonlinear polarization that emits a fourth signal field in a background-free direction. The resultant signal was combined with a collinear reference field (500 nJ, ∼100 fs) for heterodyne detection, upconverted to the visible by sum-frequency generation with a chirped 800 nm pulse, and detected using a spectrometer coupled to a CCD camera. The delay was scanned continuously between the two excitation pulses, and the resulting interferograms were Fourier transformed (resolution ∼2 cm^–1^) to obtain the excitation frequency axis in the 2DIR spectra; the detection frequency axis is obtained directly in the spectrometer. A detailed description of the technique is described in previous manuscripts.^40^

To obtain the spectral dynamics, two types of experiments were conducted: a rephasing (photon echo) and nonrephasing experiment, only differing from each other by their phase matching conditions, *k*_s,r_ = –*k*_1_ + *k*_2_ + *k*_3_ and *k*_s,nr_ = +*k*_1_ – *k*_2_ + *k*_3_ respectively. The dynamic observables obtained from these particular experiments are the vibrational lifetime, the inter- or intra-molecular vibrational redistribution time and the frequency–fluctuation correlation function, with this manuscript focusing on the latter. To obtain the FFCF, we calculate the Inhomogeneity Index (*I*(*t*_2_), eqn (1)).^15^1
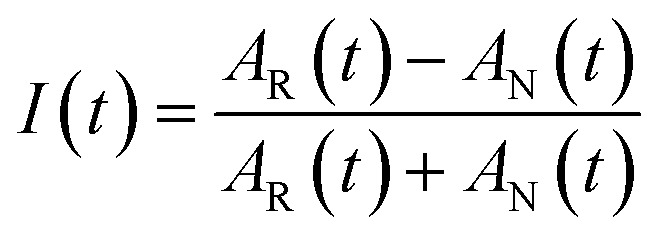



The peak amplitude from the nonrephasing (*A*_nr_) experiment is subtracted from the peak amplitude of the rephasing experiment and the difference is normalized. This procedure is repeated for each waiting time delay (*t*_2_). Since *I*(*t*_2_) is only proportional to the FFCF, we must use the following equation (eqn (2)) to calculate the FFCF:2
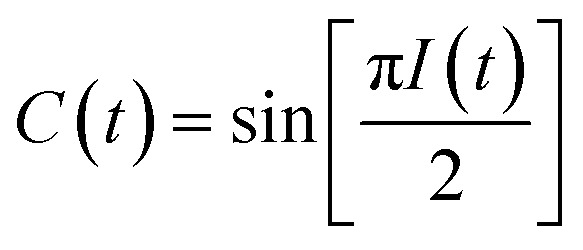



This method of measuring the FFCF has been described previously by Tokmakoff *et al.* and by us.^15^,^41^

## Conflicts of interest

There are no conflicts to declare.

## Supplementary Material

Supplementary information
