# Supplementary material for: Solvent exchange in preformed photocatalyst-donor precursor complexes determines efficiency
Source: Chem Sci. 2017 Dec 21;9(6):1527–33. doi: 10.1039/c7sc04533f (PMC5887230; doi:10.1039/c7sc04533f)
Supplement: Supplementary file 1 [file SC-009-C7SC04533F-s001.pdf]

Electronic Supplementary Material (ESI) for Chemical Science.
This journal is © The Royal Society of Chemistry 2017

Electronic Supplementary Information

Solvent Exchange in Preformed Photocatalyst-Donor Precursor Complex Determines Efficiency

Laura M. Kiefer, Kevin J. Kubarych*
[1]*Department of Chemistry, University of Michigan, 930 N. University Ave., Ann Arbor, MI 48109, United States*
*E-mail kubarych@umich.edu

Contents

S1. Complementary Preferential Solvation Studies
S2. Determination of TEOA Diffusion Coefficient in THF Solvent
S3. Estimation of Solvent Exchange Contribution to Spectral Diffusion
S4. Test of Diffusion-Limited Electron Transfer
S5. References

S1. Complementary Preferential Solvation Studies

To complement our 2DIR results, we performed $E_T(30)$ experiments by combining ratios of TEOA/Solvent with betaine-30, or Reichardt's Dye, (2,6-diphenyl-4-(2,4,6-triphenyl-1-pyridinio)phenolate), and collected UV-Vis spectra of each mixture. Betaine-30 has been used in previous preferential solvation studies, and was used here to characterize preferential solvation of triethanolamine in the solvents used in this study, dimethyl sulfox-

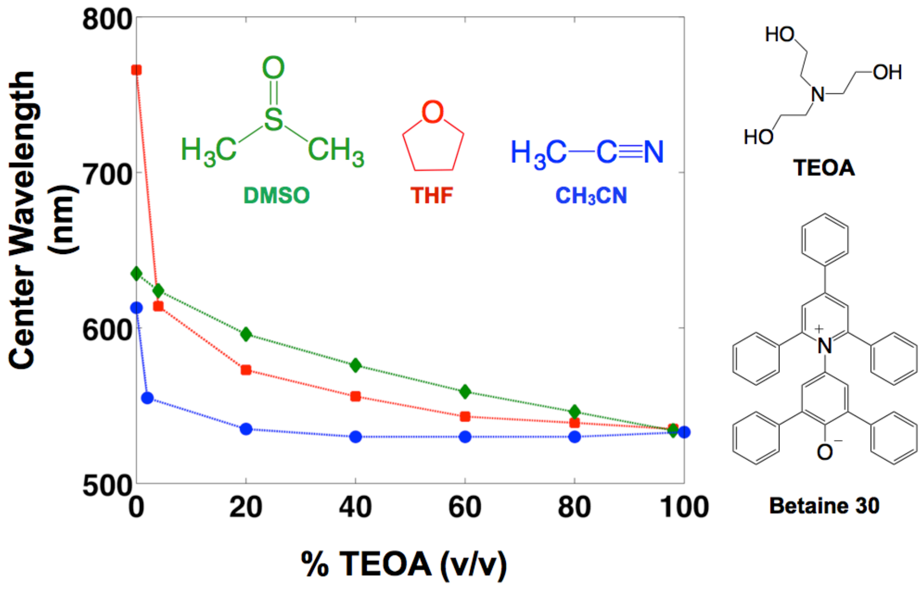

Figure S1 : Wavelengths corresponding to betaine-30 absorption maxima in the visible absorption spectra for multiple ratios of TEOA:solvent, where the solvent = DMSO, THF or CH_3CN. The figure shows the structures of the solvents and betaine-30.

ide (DMSO), tetrahydrofuran (THF) and acetonitrile (CH_3CN). Betaine-30 was selected because it is (1) widely used to characterize solvent polarity, (2) has a large ground state dipole moment that also decreases in the electronic excited state, and (3) does not exhibit charge transfer complexation as we observed in the UV-Vis spectrum of the rhenium complex. The betaine-30 dye exhibits preferential solvation as evidenced by a peak shift of the maximum visible absorption band at low concentrations of the cosolvent, in this case, the TEOA. Both betaine-30 and the rhenium complex are alike in that they both undergo bathochromic spectral shifts within a solvent (i.e. $\mu_g > \mu_e$). $E_T(30)$ values were determined using the wavelength positions of the absorption band maxima (λ_{max}) in the following equation[1]:

$$E_T(30)(kcal/mol) = \frac{28591(kcal \bullet nm \bullet mol^{-1})}{\lambda_{max}(nm)} \quad \text{(S1)}$$

The solvents used were DMSO, THF and CH_3CN. Figure S1 depicts the maximum wavelength in the visible absorption spectra for the solvent combinations.

In the solvents THF and CH_3CN, a sharp shift in the spectrum is observed at a concentration of ~2% TEOA (v/v), while in the DMSO it only shifted slightly. This is the first indication that the TEOA preferentially solvates betaine-30 in THF and CH_3CN, but not in DMSO, just as were observed in the 2DIR results. Upon further addition of TEOA, the

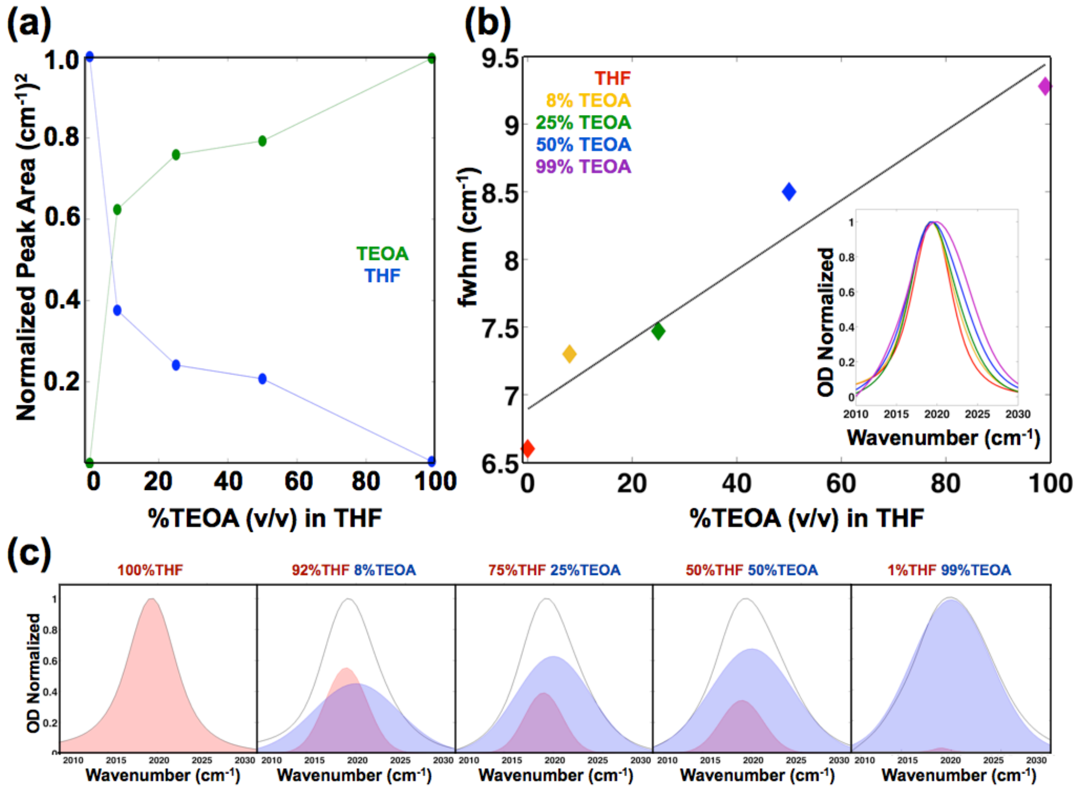

Figure S2: (a) individual peak areas of the A'(1) band of the solvent mixtures, fit to the peaks in the neat solvents, THF (blue) and TEOA (green); (b) fwhm of the A'(1) band in TEOA:THF mixtures; (c) plot of the individual gaussian fits of the A'(1) band in TEOA:THF mixtures, a plots showing fits used to obtain data in (a).

DMSO showed an essentially linear shift in the spectrum, indicating no evidence of preferential solvation. When comparing properties of the solvents to rationalize these peak shifts, we first eliminated factors that were not responsible for the observed shift. The permanent dipole moment of the TEOA (μ = 3.48 D) is comparable to those of DMSO (μ = 3.90 D) and CH_3CN (μ = 3.44 D), but much larger than that of THF (μ = 1.7 D). Nevertheless, we observe preferential solvation when $\mu_{TEOA} < \mu_{SOLVENT}$. There is not a perceived trend with the electrophilic properties (acceptor number, AN) for the three solvents: THF (AN = 8.0), CH_3CN (AN = 18.9) and DMSO (AN = 19.3). The solvents having the two lower values both exhibit preferential solvation of the TEOA, but the higher value of the two is very close to DMSO's value, and the AN must therefore be considered to contribute little, if at all. Both THF and CH_3CN have lower polarizability values (π* = 75 and 58, respectively) than DMSO (π* = 100), so this interaction may possibly influence the solvation preference. Finally, the donicities (DN = donor number) of the solvents show a similar same trend: both THF (DN = 20.0) and CH_3CN (DN = 14.1) have lower values than DMSO (DN = 29.9)[2]. Higher donor numbers correlate with stronger hydrogen bond acceptors, and these solvent DN's possibly indicate that hydrogen bonding between TEOA and DMSO may prevent preferential solvation of the solute chromophore.

Often FTIR experiments are indicators of preferential solvation through observation of a peak shift upon changes in solution composition. We conducted FTIR experiments on the mixtures, but only observe a maximum blueshift of 1 cm^{-1} of the A'(1) band upon addition of TEOA in THF. The pronounced feature is the spectral broadening, which shows a linear dependence on %TEOA (Figure S2 (b)). With a peak shift of only 1 cm^{-1} and no deviation from linearity of the fwhm vs. %TEOA, there is little indication of preferential solvation from this analysis of the FTIR results. However, we considered an equilibrium of two species, fitting each band to a sum of two gaussians with their band positions fixed by the those found in the neat solvents, (THF: 2019 cm^{-1}, and TEOA: 2020 cm^{-1}). Allowing only the spectral width and the amplitudes to vary, comparison of the the areas of each peak indicates a strong signature of preferential solvation (Figure S2 (c)). A sharp change accompanies a small increase in volume % TEOA (Figure S2 (a)). Though capable of supporting our interpretation of preferential solvation in the solvent mixtures, neither the UV-Vis nor FTIR data report any unambiguous dynamical information that may be useful for the underlying photo-catalytic mechanism.

S3. **Determination of TEOA Diffusion Coefficient in THF Solvent**

The diffusion constant of TEOA and THF in TEOA/THF (20% TEOA v/v) solution was determined using DOSY NMR. The peak intensity is given by:

$$I = I_0 \exp\left[-D\gamma^2 g^2 \delta^2 \left(\Delta - \delta/3 - \tau/2\right)\right]$$

where *D* is the diffusion constant of the species corresponding to the chemical shift analyzed, γ is the gyromagnetic ratio, *g* is the gradient strength, and Δ, δ, and τ are time delays associated with the pulse sequence (values are given below). By varying the field gradient, it is possible to extract the diffusion coefficient. Fitting the following equation:

$$\ln\left[\frac{I}{I_0}\right] = -D\gamma^2 g^2 \delta^2 \left(\Delta - \delta/3 - \tau/2\right)$$

to a line allows us to determine D since the remaining values are set in the experiment or are physical constants. The specific values are:

Δ = 150 ms

δ = 2000 μs

τ = 200 μs

γ = 26.75×10^7 T^{-1} s^{-1} (T = tesla)

g = 50 G/cm at full gradient strength (G = gauss)

1 T = 10^4 G

Figure S3 shows the measured data along with the linear fits and the fitting parameters.

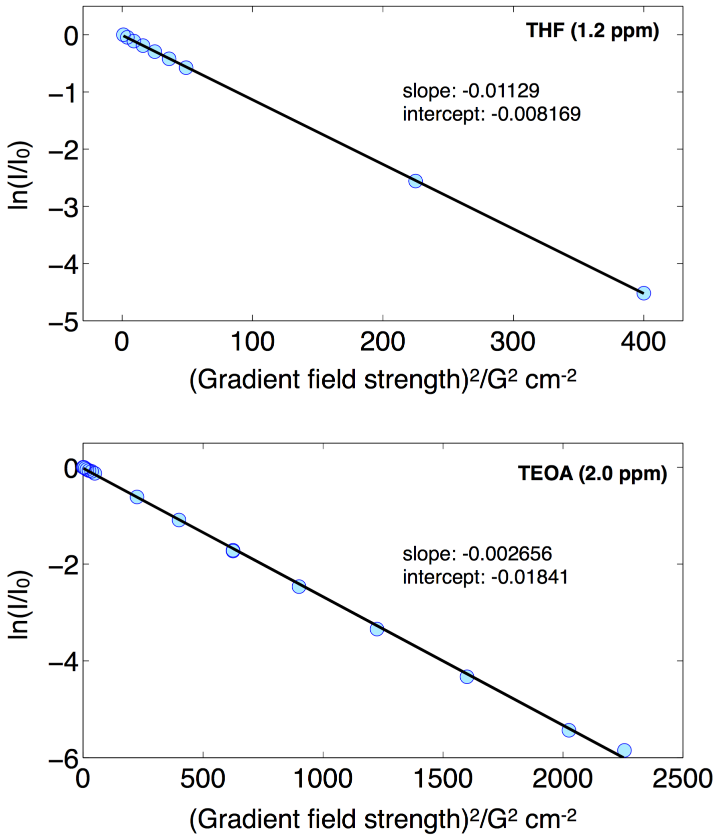

Figure S3. DOSY results and associated linear fits for a 20% (v/v) solution of TEOA in THF. (top) ln(I/I0) for the peak at a chemical shift of 1.2 ppm corresponding to THF; (bottom) ln(I/I0) for the peak at a chemical shift of 2.0 ppm corresponding to TEOA.

From the fits, we obtain D = 6.22×10^{-10} m^2/s for TEOA, and D = 2.64×10^{-9} m^2/s for THF.

S4. Estimation of Solvent Exchange Contribution to Spectral Diffusion

Solvent shell molecules always exchange, even in pure solvents. But when there is a co-solvent, a new, generally slower dynamical time scale arises due to exchange of dissimilar solvent molecule. For example, dynamic Stokes shift measurements of fluorescent molecules in mixed solvents exhibit slower solvation dynamics than in either of the neat solvents alone [3]. We have observed a similar phenomenon in a transition metal carbonyl labeled biotin ligand in H_2O/dimethyl formamide solution [4]. We can estimate the contribution of the exchange to the spectral diffusion by a simple model of the following form:

$$C(t) = w_{solv}\exp\left(-\frac{t}{\tau_{solv}}\right) + w_{exch}\exp\left(-\frac{t}{\tau_{exch}}\right) \quad \text{(S2)}$$

where w_{solv} and w_{exch} are the weights of the solvent and the exchange contributions, respectively, t_{solv} and t_{exch} are time constants for solvent and exchange dynamics, respectively. In our estimate, we fix the solvent timescale to be the average of the similar timescales we measure in the two pure solvents, which are 3.1 ps and 4.1 ps for THF and TEOA, respectively. The average is 3.6 ps. Next, we fix the timescale for exchange based on the length scale needed for solvent exchange. A computation of the molecular volume of THF using DFT [B3LYP/6-31G(d,p)] gives a solvent radius of 3.86 Å. Using this value in conjunction with the measured diffusion constant of TEOA in THF (62 $Å^2$/ns), we obtain a timescale of:

$$t = \frac{d^2}{6D} = \frac{(3.86Å)^2}{6(62Å^2/ns)} = 40.05\,ps \quad \text{(S3)}$$

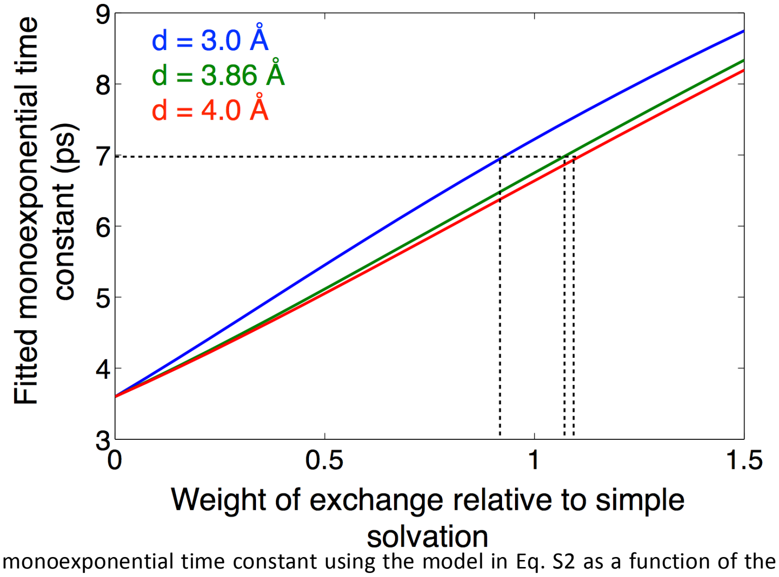

Figure S4. Fitted monoexponential time constant using the model in Eq. S2 as a function of the unknown weight of solvent exchange. The diffusion distance is not precisely known, but the molecular volume provides a strong constraint. DFT results give a solvent radius of 3.86 Å for THF. We also consider other values to show the mild sensitivity to distance (which is quadratic as see in Eq. S3). The dashed lines indicate the 20% TEOA concentration values (6.9 ps experimental time constant). A weight of 1 means the exchange and solvation contributions are equal.

Next we fit the normalized ($C(t) = 0$) correlation function model to a single exponential with an offset, in the same way that we fit our experimental data. Then we find the relative weight of solvent and exchange contributions that yield a fitted monoexponential time constant that matches the value we extract experimentally. This procedure yields a 6.9 ps apparent exponential time constant with a roughly equal weighting of the solvation and exchange contributions ($w_{solv} = 1$, $w_{exch} = 1.05$). Though we are not attempting to extract this contribution's magnitude, we note that it is reasonable, and supports the exchange origin for the slowdown of spectral diffusion observed near 20% TEOA.

Figure S4 shows the fitted monoexponential time constant as a function of the weight factor for solvent exchange for three different values of the diffusion distance. The value of 3.86 Å corresponds to the solvent radius as determined from DFT calculations. We show two other values to illustrate the lack of sensitivity to the specific distance. The dashed line indicates the experimentally determined monoexponential time constant of 6.9 ps.

S5. Test of Diffusion-Limited Electron Transfer

The picture of Collins-Kimball diffusion assisted electron transfer, which is schematically summarized in Fig. S5, depicts diffusion of quenchers to the region defined by the molecular geometry of the excited molecule. In the present study, the quencher is the

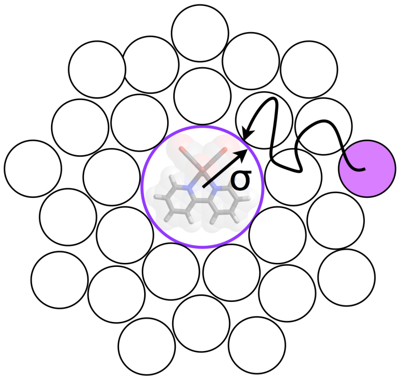

Figure S5. Cartoon indicating diffusion of an electron donor (filled purple) through solvent to contact the metastable ^{3}MLCT state of the photocatalyst, $ReCl(bpy)(CO)_3$. The reaction volume has a radius given by σ. The Collins-Kimball picture asserts that the reaction occurs with a kinetic rate constant (k_{ET}) once the donor and acceptor collide.

amine electron donor. The time-derivative of the excited state photocatalyst follows conventional chemical kinetics:

$$\frac{d}{dt}A(t) = -k_i c A(t) \tag{S4}$$

where $A(t)$ is the concentration of electron acceptors, k_i is the time-independent rate constant, and c is the concentration of electron donors (TEOA) in solution. In the classi-

cal theory of bimolecular reactions due to Smoluchowski and later expanded by Collins and Kimball (CK), reactions occur once the electron donor reaches a contact region in the immediate vicinity of the photocatalyst [5, 6]. Typically the extent of that region is somewhat adjustable, but the simplest view is that the volume is spherical with radius σ, determined by the volume of the photocatalyst. In the case of highly viscous solution, the donor may linger in the vicinity of the photocatalyst, effectively enlarging the reaction layer. In the treatment of CK, the radius of the reaction volume *R* is simply set equal to σ.

Collins and Kimball derived an expression for the time-independent rate constant (k_i), which has been shown subsequently to be reliable for low-viscosity reaction solutions. The expression is:

$$\frac{1}{k_i} = \frac{1}{k_{ET}} + \frac{1}{4\pi RD} \tag{S5}$$

where k_{ET} is the kinetic rate constant for electron transfer and *D* is the diffusion constant of the electron donor in the reaction solution. It is evident from this formula that there is a maximum rate constant when the intrinsic electron transfer is significantly faster than the motional diffusion contribution.

For the purposes of comparing directly to our time-domain experiments, we rewrite the rate constant as:

$$k_{obs} = k_i c = c\left(\frac{1}{k_{ET}} + \frac{1}{4\pi RD}\right)^{-1} \tag{S6}$$

$$\tau_{obs} = 1/k_{obs} = \frac{1}{c}\left(\frac{1}{k_{ET}} + \frac{1}{4\pi RD}\right) \tag{S7}$$

For infinite intrinsic k_{ET}, the ET time is given only by diffusion into the reaction volume:

$$\tau_{\min} = \frac{1}{4\pi cRD} \tag{S8}$$

A finite k_{ET} would simply lead to a slower overall ET time scale. A quantitative estimate of this minimum reaction time can be made using the following values: *c* = 1.5 M, *R* = 4.0 Å, *D* = 62 $Å^2$/ns. The radius is chosen from the molecular volume computed using DFT, which we found to be 168.111 (cm^3/mol), which is equal to 279.2 $Å^3$ for one molecule. The units of concentration must be adapted to the formula, which needs to give units of time. *RD* has units of volume, and *c* has units of mol L^{-1}. 1 mol L^{-1} is 6.02×10^{23} molecules/(1000 cm^3), and 1 cm^3 = 10^{24} $Å^2$. Therefore, 1 M is 6.02×10^{-4} molecules/$Å^3$. Putting these values together we find a minimum time for the diffusion limited ET process to be:

$$\tau_{\min} = \left[4\pi \left(1.5 \times 6.02 \times 10^{-4} \, molecules / \mathring{A}^3 \right) \left(4\mathring{A} \right) \left(62\mathring{A}^2 ns^{-1} \right) \right]^{-1} = 0.355 ns \qquad \text{(S9)}$$

or 355 ps. To achieve a time scale that is equal to our measured value of roughly 70 ps would require a reaction volume with radius of 20.3 Å, which is unphysically large. Moreover, any steric, orientational, or site-specific encounter requirements will necessarily reduce the rate of ET, further supporting the conclusion that we observe non-diffusive ultrafast electron transfer in the present Re/TEOA/THF photocatalyst system.

S5. References

1 C. Reichardt, *Chem. Rev.*, 1994, **94**, 2319-2358.
2 V. Gutmann, *Coord. Chem. Rev.*, 1976, **18**, 225-255.
3 N. Agmon, *J. Phys. Chem. A*, 2002, **106**, 7256-7260.
4 J. A. Dunbar, E. J. Arthur, A. M. White and K. J. Kubarych, *J. Phys. Chem. B*, 2015, **119**, 6271-6279.
5 V. S. Gladkikh, A. I. Burshtein, H. L. Tavernier and M. D. Fayer, *J. Phys. Chem. A*, 2002, **106**, 6982-6990.
6 F. C. Collins and G. E. Kimball, *J. Coll. Sci.*, 1949, **4**, 425-437.
